# Supplementary material for: The genome sequence of the Little Snipefly, Chrysopilus asiliformis (Preyssler, 1791)
Source: Wellcome Open Res. 2025 Jul 4;10:329. [Version 1] doi: 10.12688/wellcomeopenres.24326.1 (PMC12371324; doi:10.12688/wellcomeopenres.24326.1)
Supplement: Peer review history

# Reviewer report: Reviewer response for version 1

**DOI:** 10.21956/wellcomeopenres.26827.r127408

**Published:** 2025-08-21

**Authors:** Daniela H Palmer Droguett

**Affiliations:** 1 The University of Texas at Arlington, Arlington, Texas, USA

The study presents a genome assembly and annotation for the Little Snipefly ( *Chrysopilus asiliformis*). The genome assembly is 429.05 Mb in length, and 99.58% of the assembly is contained in 5 major scaffolds. The assembly contains 95.3% complete BUSCOs based on the diptera_odb10 set. The annotation includes 14,233 protein-coding genes. Based on read coverage metrics, females appear to be the homogametic sex in this species.

 A few notes that would strengthen the manuscript:

1. Are there karyotype data that can corroborate the 5 major scaffolds of the assembly?
2. Verification of female homogamety and identification of the sex chromosomes incorporating data from both sexes would be ideal.
3. Was the specimen used for RNA-seq also a female? The methods don’t specify.
4. Average transcript length is nearly 15kb, which seems quite high. Can the authors please clarify why this might be?

Are sufficient details of methods and materials provided to allow replication by others?

Yes

Is the rationale for creating the dataset(s) clearly described?

Yes

Are the datasets clearly presented in a useable and accessible format?

Yes

Are the protocols appropriate and is the work technically sound?

Yes

Reviewer Expertise:

Evolution, genomics, bioinformatics

I confirm that I have read this submission and believe that I have an appropriate level of expertise to confirm that it is of an acceptable scientific standard.

# Reviewer report: Reviewer response for version 1

**DOI:** 10.21956/wellcomeopenres.26827.r128707

**Published:** 2025-08-12

**Authors:** Cheng Sun

**Affiliations:** 1 Capital Normal University, Beijing, Beijing, China

The current study presents a chromosome-level genome sequence for the Little Snipefly, *Chrysopilus asiliformis*. The genome sequence has a total length of 429 Mb, with 5 pseudomolecules. The mitochondrial genome has also been assembled, with a length of 16.38 kilobases. Genome annotation identified 14,233 protein-coding genes.

 1) There was a typo in this paper. "chromosomal-level" should be "chromosomal-level "? 

 2) More information on Little Snipefly, *Chrysopilus asiliformis *should be provided.

Are sufficient details of methods and materials provided to allow replication by others?

Yes

Is the rationale for creating the dataset(s) clearly described?

Yes

Are the datasets clearly presented in a useable and accessible format?

Yes

Are the protocols appropriate and is the work technically sound?

Yes

Reviewer Expertise:

Genome evolution

I confirm that I have read this submission and believe that I have an appropriate level of expertise to confirm that it is of an acceptable scientific standard.
